# Risk factors for *Clostridioides difficile* infection caused by ribotype 027 strains in the Veterans Affairs Healthcare System: a matched case-control study

**DOI:** 10.1186/s13756-025-01571-0

**Published:** 2025-05-16

**Authors:** Sandra Y. Silva, Brigid M. Wilson, Curtis J. Donskey

**Affiliations:** 1 Department of Population and Quantitative Health Science, Case Western Reserve University School of Medicine, Cleveland, OH USA; 2 Geriatric Research Education and Clinical Center, Louis Stokes Veterans Affairs Medical Center, 10701 East Blvd., Cleveland, OH 44106 USA; 3 Department of Medicine, Case Western Reserve University School of Medicine, Cleveland, OH USA

**Keywords:** *Clostridioides difficile*, Ribotype 027, Stewardship, Fluoroquinolones, Risk factors

## Abstract

**Background:**

In case-control studies, a variety of factors have been associated with *Clostridioides difficile* infection (CDI) due to the epidemic ribotype 027 strain. However, many studies have been limited due to small sample size and inclusion of only one facility.

**Methods:**

Using a nationwide cohort of hospitalized patients in the Veterans Affairs (VA) Healthcare System, we conducted a retrospective, 1:3 matched case-control study of patients with CDI due to the ribotype 027 strain versus non-027 strains from October 1, 2008, to September 30, 2020. Controls were matched to cases by health care facility and year of diagnosis. Multivariate logistic regression was used to identify risk factors for CDI due to the 027 strain.

**Results:**

A total of 3,353 cases were matched to 10,059 controls in 84 VA facilities. CDI due to the ribotype 027 strain was independently associated with prior macrolide or fluoroquinolone exposure, decreased functional capability, methicillin-resistant *Staphylococcus aureus* nasal colonization, age >65 years, white blood cell count >11,000 cells/mm^3^, and serum albumin < 3.5 g/dl.

**Conclusion:**

Antimicrobial stewardship interventions focused on fluoroquinolones and macrolides could be beneficial in reducing the risk for infection due to the ribotype 027 *C. difficile* strain. Several other factors could potentially be used to identify patients at increased risk for CDI due to the ribotype 027 strain, but further studies are needed to assess their utility in clinical settings.

## Introduction

In the early 2000 s, a previously uncommon *Clostridioides difficile* strain termed ribotype 027 acquired high-level resistance to fluoroquinolone antibiotics and caused large outbreaks in North America and Europe [1, 2]. Selective pressure exerted by fluoroquinolone use was identified as a risk factor for infection with 027 strains [3–6], and in some studies, reductions in fluoroquinolone use were associated with reductions in CDI due to the 027 strain [7–11]. Various other factors have been associated with infections due to the 027 strain in case-control studies, including cephalosporin or macrolide exposure, proton pump inhibitor use, advanced age, nursing home residence, hematologic malignancy, and duration of hospitalization [4, 12–16]. However, the conclusions from many of these studies have been limited due to small sample size and inclusion of only one facility.

The Veterans Health Administration (VHA) is America’s largest integrated healthcare system, providing care at 171 medical centers and 1,113 outpatient sites. The VA system provides a unique opportunity to study the ribotype 027 *C. difficile* strain on a large scale because many VA hospitals use molecular tests that distinguish 027 from other strains and both individual patient and facility-level data can be analyzed [17, 18]. In a previous study of 55 VA hospitals, we reported a 55% reduction in the proportion of CDI cases due to the 027 strain between 2011 and 2018 and demonstrated a significant effect of facility-level fluoroquinolone use on infections due to the 027 strain [17]. Here, we conducted a case-control study to identify patient-level risk factors for infection with the 027 strain.

## Materials and methods

### Setting and study population

The Louis Stokes Cleveland VA Medical Center Institutional Review Board approved the study protocol. The VA Informatics and Computing Infrastructure was used to obtain data from the Corporate Data Warehouse, a central VA Healthcare System data repository containing administrative, clinical, laboratory, and pharmacy data linked using unique patient identifiers [17]. We generated a nationwide cohort of adult patients with one or more hospital admissions during the study period and a laboratory diagnosis of CDI meeting the criteria for classification as an episode of CDI between October 1, 2008, and September 30, 2020. CDI cases were identified based on positive *C. difficile* results by polymerase chain reaction (PCR) for toxigenic *C. difficile* or enzyme immunoassay (EIA) for toxin. Patients with positive CDI tests and simultaneous testing for the 027 strain were included in the study cohort. Testing for the 027 strain was performed either using Xpert *C. difficile*/Epi Assay (Cepheid, Sunnyvale, CA) or the Verigene *C. difficile* test (Luminex, Austin, TX). During the study period, the national VA infectious diseases program recommended that all patients diagnosed with CDI be place in contact precautions.

Cases were defined as patients testing positive for CDI with the 027 strain and controls were defined as patients testing positive for CDI but negative for the 027 strain. Three controls were selected for each case. Cases and controls were matched by VA health care facility and fiscal year to identify matched pairs with the same location- and time-specific prevalence of 027 strain. Cases of CDI with previous episodes in the past year were excluded prior to matching. We obtained patient-level data on demographics, comorbid illnesses, Charlson Comorbidity Index Score, Braden Score (i.e., a standardized score used to assess risk for pressure ulcers with lower scores indicating increased risk), gastrointestinal procedures, laboratory data, and medication use, including systemic antibiotics and acid suppressants. CDI cases were classified as community-associated versus healthcare-associated based on standard surveillance definitions [19]. Table 1 provides definitions of the variables included in the analysis.

**Table 1:** Variable definitions

Variable	Description
Age	Calculated based on the patient’s date of birth and date of positive test for *C. difficile.*
Body Mass Index (BMI)	Patient’s weight (kg) divided by the square of the patient’s height (meters).
Race	White (Caucasian) or non-white (American Indian or Alaska Native; Asian; black or African American; Native Hawaiian or Other Pacific Islander).
Charlson Comorbidity Index	Weighted index to predict the risk of death in patients with specific comorbidities based on the International Classification of Diseases (ICD) diagnosis code, ninth and tenth revisions ICD-9, ICD-10).
Braden Score	Measure of functional capabilities to predict the risk of developing facility-acquired pressure ulcer/injury. Most recent score within 90 days prior to the positive *C. difficile* test.
Comorbidities	History of diabetes mellitus, cancer, cirrhosis, inflammatory bowel disease (ulcerative colitis or Crohn’s disease). Based on ICD-9 and ICD-10.
Gastrointestinal Procedures	Endoscopy or gastric tube placement within 15 days prior to a positive *C. difficile* test.
Nasal colonization of methicillin-resistant Staphylococcus aureus, (MRSA)	Nasal-swab screening for methicillin-resistant *Staphylococcus aureus* (MRSA) within 90 days prior to a positive *C. difficile* test.
Serum creatinine and peripheral white blood cell count	Most recent test within 90 days prior to a positive *C. difficile* test.
Serum albumin	Most recent test within 180 days prior to a positive *C. difficile* test.
High Risk Antibiotics	Defined as days of therapy (DOT) of high-risk antibiotics for CDI (carbapenems, macrolides, clindamycin, fluoroquinolones, piperacillin-tazobactam, 2nd, 3rd, and 4 th generation cephalosporins) during the previous 90 days prior a positive *C. difficile* test. Piperacillin-tazobactam was recorded in inpatient services only.
Proton pump inhibitors and histamine H2-receptor antagonists	One or more oral/intravenous doses within 90 days before a positive *C. difficile* test.
Epidemiological Classification	Community-associated: CDI symptom onset in the community or <4 days from admission (day of admission being day 1), provided that symptom onset was >12 weeks after the last discharge from a healthcare facility. Healthcare-associated: CDI symptom onset ≥4 days after admission to a healthcare facility, with day of admission being day 1, or in the community or <4 days from admission provided that symptom onset was <4 weeks after the last discharge from a healthcare facility.

### Statistical analysis

Missing data occurred in 6 variables. For these variables, the percentages of cases and controls with missing data were similar: nasal colonization by methicillin-resistant *Staphylococcus aureus* (MRSA) (cases, 18%; controls, 17%), Braden Scale (16%; 17%), serum white blood cell (WBC) count (16%; 15%), creatinine (14%; 11%), albumin (12%; 10%), body mass index (BMI) (2%; 3%). Missing data were imputed to prevent potential loss of statistical precision and reduce the probability of bias in the logistic regression analysis when using only complete patient data. Predictive means matching was used to generate the imputed values.

Univariate conditional logistic regression was used to identify individual risk factors potentially associated with infection with the 027 strain, and conditional logistic regression models were used to assess the independence of effects. The estimates were reported in odds ratios (ORs) with 95% confidence intervals (CIs). All the clinical variables listed in Table 1 were included in multivariable logistic regression models. Total high-risk antibiotic days of exposure and individual high-risk antibiotic days of exposure were included in the multivariable logistic regression model.

We conducted a subgroup analysis by epidemiological classification. Healthcare-associated and community-associated cases were matched in a 1:3 ratio with their respective controls; the same statistical approach was used to compare 027 cases and controls. Data analysis was performed with RStudio Inc. (Version 2022.02.3, Boston, MA, USA) for MAC (https://www.r-project.org/), using the R packages *mice*, Matching, MASS, Epi, survival.

## Results

### Study participants

**Fig. 1:**
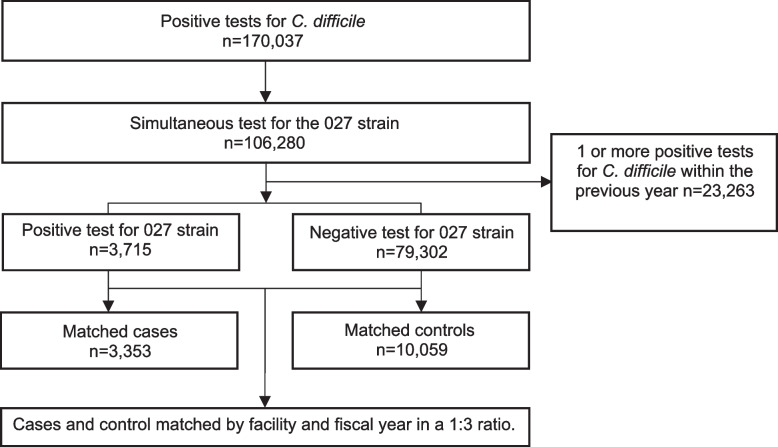
Flow diagram of study participants

A flow diagram for the study participants is shown in Fig. 1. Of 106,280 cases from 84 facilities testing positive for *C. difficile* with a simultaneous test for the 027 strain, 83,017 did not have a prior *C. difficile* test within the past year. Of these 83,017 CDI cases, 3,715 (4.5%) tested positive for the 027 strain and 79,302 (95.5%) tested negative. We successfully matched without replacement 3,353 CDI cases due to 027 strains in 2735 patients with 10,059 control cases due to non-027 strains in 8907 patients. Of the 13,412 CDI cases included in the analysis, 10,943 (81.6%) were in the hospital at the time of diagnosis, 1,409 (10.5%) were in a long-term care facility, 738 (5.5%) were outpatients, and 322 (2.4%) were in rehabilitation.

### Risk factors for CDI with the 027 strain

Table 2 provides a comparison of the demographic and clinical characteristics of the case patients with CDI due to the 027 strain versus controls infected with non-027 strains. By univariable analysis, patients infected with 027 strains were significantly more likely to be >65 years-old and male and had higher Charlson Comorbidity Index Scores and lower Braden Scores. Diabetes mellitus and MRSA colonization were more common in case patients, whereas inflammatory bowel disease was more common in control patients. Patients infected with 027 strains had higher serum creatinine levels, higher peripheral WBC counts, and lower albumin levels than controls. Patients infected with 027 strains were significantly more likely to have healthcare-associated CDI (65.1% versus 54.3%) and to receive high-risk antibiotics within 90 days before the diagnosis of CDI (66.1% versus 55.2%). Among the high-risk antibiotic classes, case patients were more likely than control patients to have received fluoroquinolones (51% versus 46 %), third-generation cephalosporins (35% versus 32%), and macrolides (19% versus 11%).

**Table 2:** Characteristics of patients with *Clostridioides difficile* infection (CDI) due to the ribotype 027 strain (Cases) versus CDI due to non-027 strains (Controls)

Characteristics	Cases (*n*=3,353)	Controls (*n*=10,059)	OR (95% CI)	Univariate Analysis *P* Value
Gender male (%)	3191 (95.2)	9412 (93.6)	1.35 (1.14–1.61)	0.001
Age >65 years old (%)	2413 (72.0)	6308 (62.7)	1.53 (1.41–1.66)	<0.001
BMI, kg/m2 [Mean (SD)]	27.27 (7.61)	27.76 (7.39)	0.99 (0.98–0.99)	0.001
Race, White (%)	2760 (82.3)	8204 (81.6)	1.06 (0.95–1.17)	0.300
CCIS [Mean (SD)]	4.27 (3.26)	3.91 (3.25)	1.04 (1.02–1.04)	0.001
Braden Scale (%)				
No Risk	1617 (48.2)	5806 (57.7)	Ref	
Mild	1257 (37.5)	3211 (31.9)	1.43 (1.31–1.56)	0.000
Moderate	273 (8.1)	619 (6.2)	1.62 (1.39–1.89)	0.000
High	206 (6.1)	423 (4.2)	1.79 (1.5–2.14)	0.000
Inflammatory Bowel Disease (%)	56 (1.7)	389 (3.9)	0.42 (0.31–0.56)	<0.001
Diabetes Mellitus (%)	1456 (43.4)	4019 (40.0)	1.16 (1.07–1.25)	0.000
Cancer (%)	903 (26.9)	2622 (26.1)	1.05 (0.96–1.14)	0.323
Cirrhosis (%)	234 (7.0)	648 (6.4)	1.09 (0.93–1.27)	0.276
Gastrointestinal procedure (%)	67 (2.0)	230 (2.3)	0.87 (0.66–1.15)	0.327
Nasal colonization MRSA (%)	528 (15.7)	1038 (10.3)	1.66 (1.48–1.86)	<0.001
Serum creatinine, >1.3 mg/dL (%)	1484 (44.3)	4019 (40.0)	1.2 (1.1–1.29)	<0.001
Peripheral WBC count, ≥11,000/mm (%)	1641 (48.9)	3618 (36.0)	1.71 (1.58–1.85)	<0.001
Serum albumin, <3.5 g/dL (%)	2496 (74.4)	6435 (64.0)	1.69 (1.55–1.86)	<0.001
PPIs (%)	1911 (57.0)	5783 (57.5)	0.98 (0.9–1.06)	0.610
H2RAs (%)	598 (17.8)	1709 (17.0)	1.06 (0.96–1.18)	0.257
High-risk antibiotic time of exposure within 90 days (%)				
None	1137 (33.9)	4502 (44.8)		
≤5 days	872 (26.0)	2511 (25.0)	1.39 (1.25–1.54)	<0.001
6–14 days	962 (28.7)	2222 (22.1)	1.75 (1.58–1.93)	<0.001
≥14 days	382 (11.4)	824 (8.2)	1.90 (1.65–2.18)	<0.001
Epidemiological Classification				
Community-associated	1001 (29.9)	4016 (39.9)	Ref	
Healthcare-associated	2182 (65.1)	5458 (54.3)	1.64 (1.5–1.79)	<0.001
Undetermined	170 (5.1)	585 (5.8)	1.14 (0.94–1.38)	0.195

Data are no. (%) of patients, unless otherwise specified

*Abbreviations*: *OR* odds ratio, *CCIS* Charlson Comorbidity Index Score, *BMI* body mass index, *MRSA* methicillin-resistant *Staphylococcus aureus*, *WBC* White blood cells, *PPI* Proton pump inhibitors, *H2RAs* Histamine H2-receptor antagonists

Table 3 shows the results of the multivariable conditional logistic regression analysis.

**Table 3:** Multivariable conditional logistic model to identify risk factors for *Clostridioides difficile* infection (CDI) due to the ribotype 027 strain

Characteristics	OR	Lower.95	Upper.95	p
Gender male	1.02	0.85	1.23	0.839
Age > 65 years old	1.28	1.16	1.41	<0.001
BMI, kg/m2	0.99	0.99	1.00	0.055
Race, white	1.04	0.93	1.16	0.536
CCIS	1.01	0.99	1.03	0.440
Braden Scale				
No Risk	Ref			
Mild	1.19	1.09	1.31	<0.001
Moderate	1.28	1.09	1.51	0.003
High	1.31	1.09	1.58	0.004
Inflammatory Bowel Disease	0.53	0.40	0.71	<0.001
Diabetes Mellitus	1.02	0.93	1.13	0.632
Cancer	0.97	0.87	1.08	0.572
Cirrhosis	1.18	1.00	1.39	0.052
Gastrointestinal procedure	0.73	0.55	0.97	0.032
Nasal colonization MRSA	1.33	1.18	1.50	<0.001
Serum creatinine, >1.3 mg/dL	1.03	0.94	1.13	0.503
Peripheral WBC count, ≥11k/mm	1.48	1.36	1.61	<0.001
Serum albumin, < 3.5 g/dL	1.33	1.20	1.47	<0.001
PPIs	0.86	0.79	0.93	<0.001
H2RAs	0.94	0.84	1.05	0.282
High Risk Antibiotics time of exposure within 90 days				
None	Ref			
≤ 5 days	1.13	1.01	1.26	0.036
6–14 days	1.38	1.23	1.55	<0.001
≥ 14 days	1.56	1.34	1.82	<0.001
*Epidemiological Classification*				
Community associated	Ref			
Health Care Associated	1.24	1.12	1.38	<0.001
Undetermined	1.34	1.10	1.63	0.004

*Abbreviations*: *OR* odds ratio, *BMI* body mass index, *CCIS* Charlson Comorbidity Index Score, *MRSA* methicillin-resistant *Staphylococcus aureus*, *WBC* White blood cells, *PPI* Proton pump inhibitors, *H2RAs* Histamine H2-receptor antagonists

High-risk antibiotic therapy was independently associated with infection due to 027 strains with a dose-dependent increase in risk with increasing days of exposure. Infection with the 027 strain was also independently associated with reduced Braden Score, indicating an increased risk of pressure ulcers, age greater than 65 years old, MRSA colonization, peripheral white blood cell (WBC) ≥11,000 cells per mm^3^, serum albumin <3.5 g/dl, and classification as healthcare associated.

**Fig. 2:**
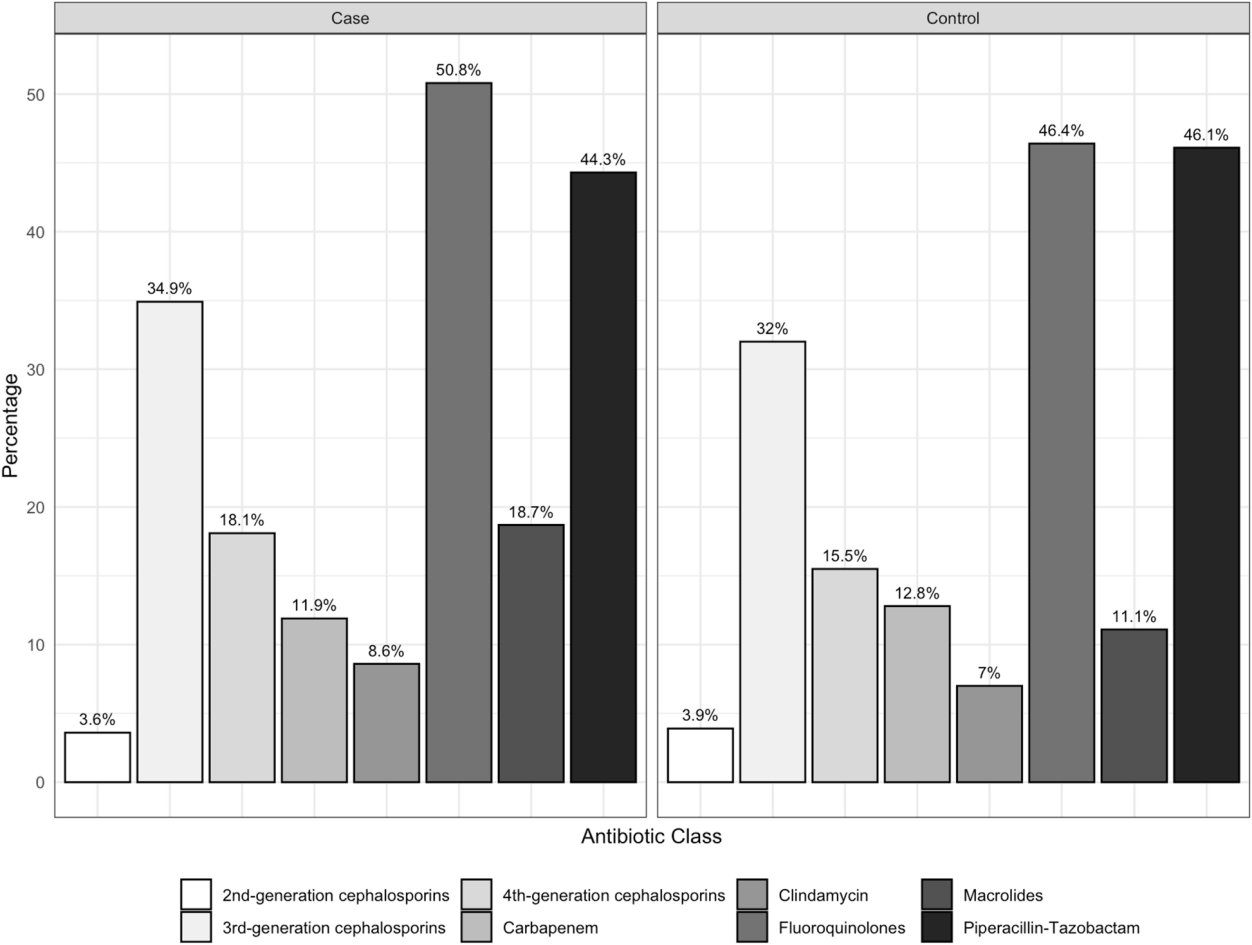
Percentages of prior exposure to different classes of high-risk antibiotics among case patients with *Clostridioides difficile* infection (CDI) due to the ribotype 027 strain and control patients with CDI due to non-027 strains

Figure 2 shows the percentages of prior exposure to different classes of high-risk antibiotics among case patients with CDI due to the ribotype 027 strain and control patients with CDI due to non-027 strains. In comparison to patients infected with non-027 strains, patients infected with the ribotype 027 strain were significantly more likely to have received prior treatment with fluoroquinolones (50.8% versus 46.4%; *P*=0.026) and macrolides (18.7% versus 11.1%); *P*= <0.001).

**Fig. 3:**
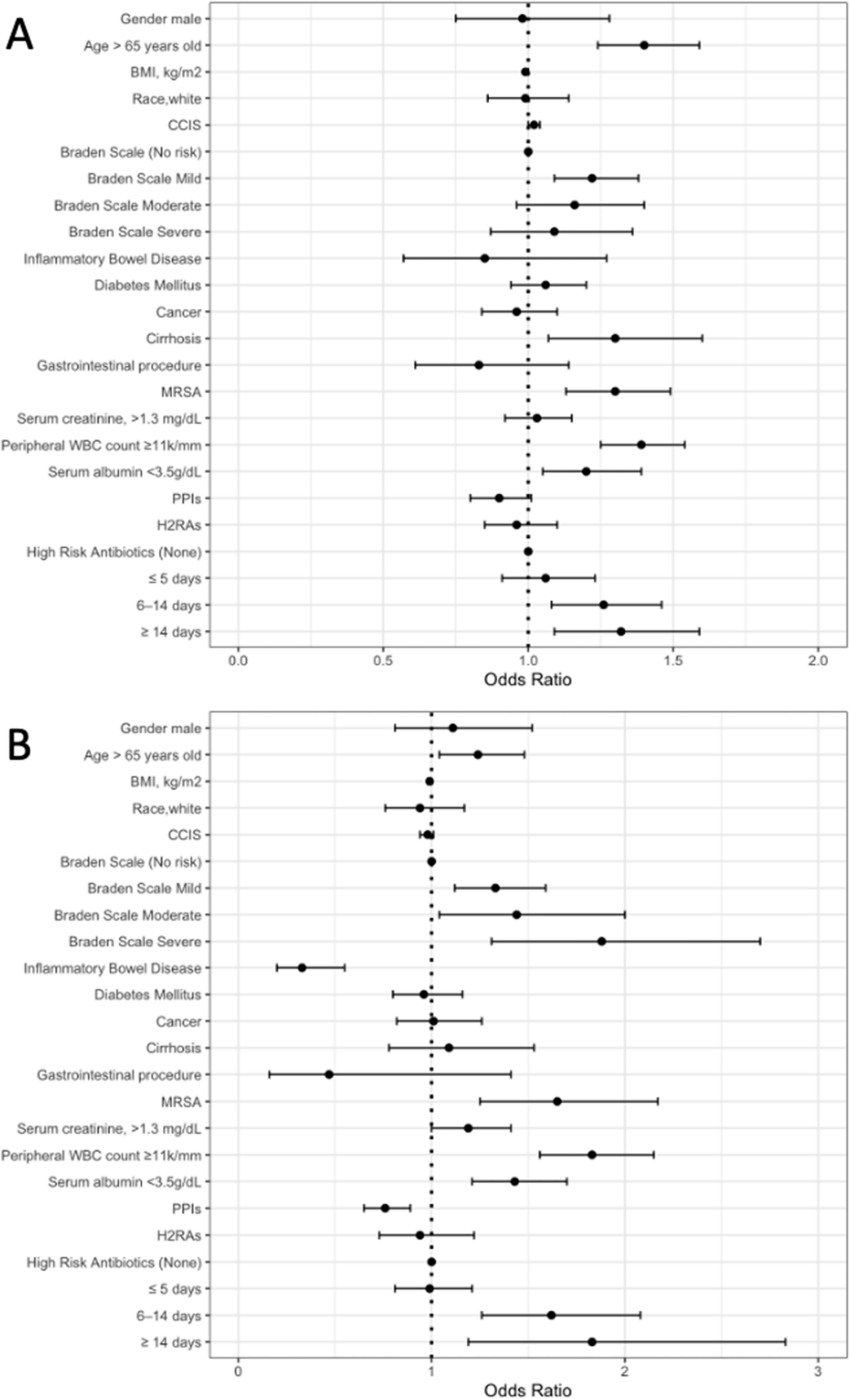
Odd ratios and 95% confidence intervals (CI) of independent risk factors associated with *Clostridioides difficile* infection (CDI) due to the ribotype 027 strain for healthcare-associated (**A**) and community-associated CDI

Because infection with ribotype 027 strains was significantly more common in patients with healthcare-associated CDI, we performed a subgroup analysis of patients with healthcare-associated versus community-associated CDI. As shown in Fig. 3, the multivariable analysis for subgroups of healthcare-associated and community-associated CDI cases yielded similar results. For both healthcare-associated and community-associated CDI, infection with the 027 strain was independently associated increased duration of high-risk antibiotics, reduced Braden Score, age greater than 65 years old, MRSA colonization, and peripheral WBC >11,000 cells per mm3.

## Discussion

The ribotype 027 strain of *C. difficile* caused large outbreaks in North America and Europe in the early 2000s [1, 2]. Although the overall prevalence of the ribotype 027 strain has decreased in recent years, it remains a common cause of CDI [17, 18]. Many previous studies that examined risk factors for infection with the ribotype 027 strain have been limited due to the small sample size and inclusion of only one facility. In the current study, we analyzed a large nationwide sample of patients with CDI to identify the risk factors for infection caused by the 027 strain.

Our findings suggest that antimicrobial stewardship interventions focused on fluoroquinolones and macrolides could be beneficial in reducing the risk for infection due to ribotype 027 *C. difficile* strains. CDI due to the ribotype 027 strain was independently associated with exposure to high-risk antibiotics within 90 days with a dose-dependent increase in risk with increasing days of exposure. However, subgroup analysis demonstrated that fluoroquinolones and macrolides were the only high-risk agents that were independently associated with infection with the ribotype 027 strain. These results are consistent with previous studies that identified fluoroquinolone use as an important risk factor for the development of CDI due to the 027 strain [3, 4, 6, 7, 12, 17, 18, 20]. Macrolides have not been identified as a common risk factor for the ribotype 027 strain. However, Wieczorkiewicz et al. [4] reported that macrolide exposure as an independent risk factor for CDI due to the ribotype 027 strain and noted that many 027 strains exhibited high-level resistance to these agents (defined as azithromycin MIC ≥64 μg/mL).

In addition to antibiotic exposure, CDI due to the ribotype 027 strain was associated with low Braden score, MRSA colonization, age >65 years, white blood cell count >11,000 cells/mm^3^, and serum albumin < 3.5 g/dl. Many of these factors have been associated with higher mortality and severity of illness in CDI [4, 13, 19, 21, 22]. Thus, it is possible that infection with the ribotype 027 strain is not directly responsible for the poor outcomes associated with these strains, but rather the fragile health status of patients infected with these strains. Other studies have also reported a relationship between older age and the development of CDI due to 027 strains [12, 13, 15, 20]. The Braden scale is an excellent tool for predicting frailty and mortality associated with several diseases [21, 22]. A previous cohort study conducted in the VA healthcare system demonstrated an association between frailty and CDI [23]. Although these factors could potentially be used to identify patients at increased risk for CDI due to 027 strains, they may have limited usefulness in clinical settings given the modest differences between the 027 and non-027 groups.

We did not find that PPI use was associated with CDI due to the ribotype 027 strain. One previous small study reported an association between PPI use and infection with the ribotype 027 strain [14]. However, several other studies have not reported an association between PPIs and CDI due to the 027 strain [4, 15, 16].

Our study has several limitations. First, the study was conducted in the VA healthcare system using only VA data sources. The findings may not be generalizable to non-VA healthcare settings, and we might have missed some medical care and medications received outside the VA system. Second, CDI cases were identified based on positive PCR for toxigenic *C. difficile* with or without a positive EIA for free toxin. Thus, some cases classified as CDI might have been colonization rather than true infection [24]. Third, although the commercial molecular tests detect the 027 strain, they may also detect other strains that have similar genetic findings within the *C. difficile* pathogenicity locus and binary toxin CDT gene *cdtB* [25, 26]. Thus, we cannot confirm that all the infections classified as being due to the 027 strain were due to that strain type. Finally, we do not have susceptibility testing results for the 027 and non-027 isolates causing CDI in this study. The Clinical Laboratory Standards Institute (CLSI) has not established specific breakpoints for fluoroquinolones against *C. difficile*. Based on CLSI breakpoints for anaerobic bacteria, ribotype 027 isolates have high rates of resistance to fluoroquinolones (resistance and high-level resistance defined as MIC ≥8 and ≥64 µg/mL, respectively), but some non-027 isolates also exhibit resistance [4, 6]. In a previous study in 1 of the VA facilities that included susceptibility testing and ribotyping, 95% of ribotype 027 isolates were moxifloxacin-resistant versus only 6% of non-027 isolates [27].

## Conclusion

We found that CDI caused by ribotype 027 strain was associated with fluoroquinolone and macrolide exposure with a dose-dependent increase in risk with increasing days of exposure. Previous studies have demonstrated that fluoroquinolones are often used unnecessarily and for longer than recommended durations [6, 28]. Thus, antimicrobial stewardship interventions to reduce unnecessary use of fluoroquinolones and macrolides and ensure that the duration of treatment is appropriate could be beneficial in reducing the risk for infection due to the ribotype 027 strain.

## Data Availability

The datasets used and/or analyzed during the current study are available from the corresponding author on reasonable request.

## Abbreviations

CDI: *Clostridioides** difficile* infection
VA: Veterans Affairs
VHA: Veterans Health Administration
WBC: White blood cell
OR: Odds ratio
CI: Confidence interval
EIA: Enzyme immunoassay
CCIS: Charlson Comorbidity Index Score
BMI: Body mass index
PPI: Proton pump inhibitor
H2RA: Histamine H2-receptor antagonist
